# Supplementary material for: The causal relationship between the human gut microbiota and pyogenic arthritis: a Mendelian randomization study
Source: Front Cell Infect Microbiol. 2024 Nov 26;14:1452480. doi: 10.3389/fcimb.2024.1452480 (PMC11629706; doi:10.3389/fcimb.2024.1452480)
Supplement: Supplementary file 1 [file DataSheet1.zip › mendelian test/ebi-a-GCST90027667.csv_forest.pdf]

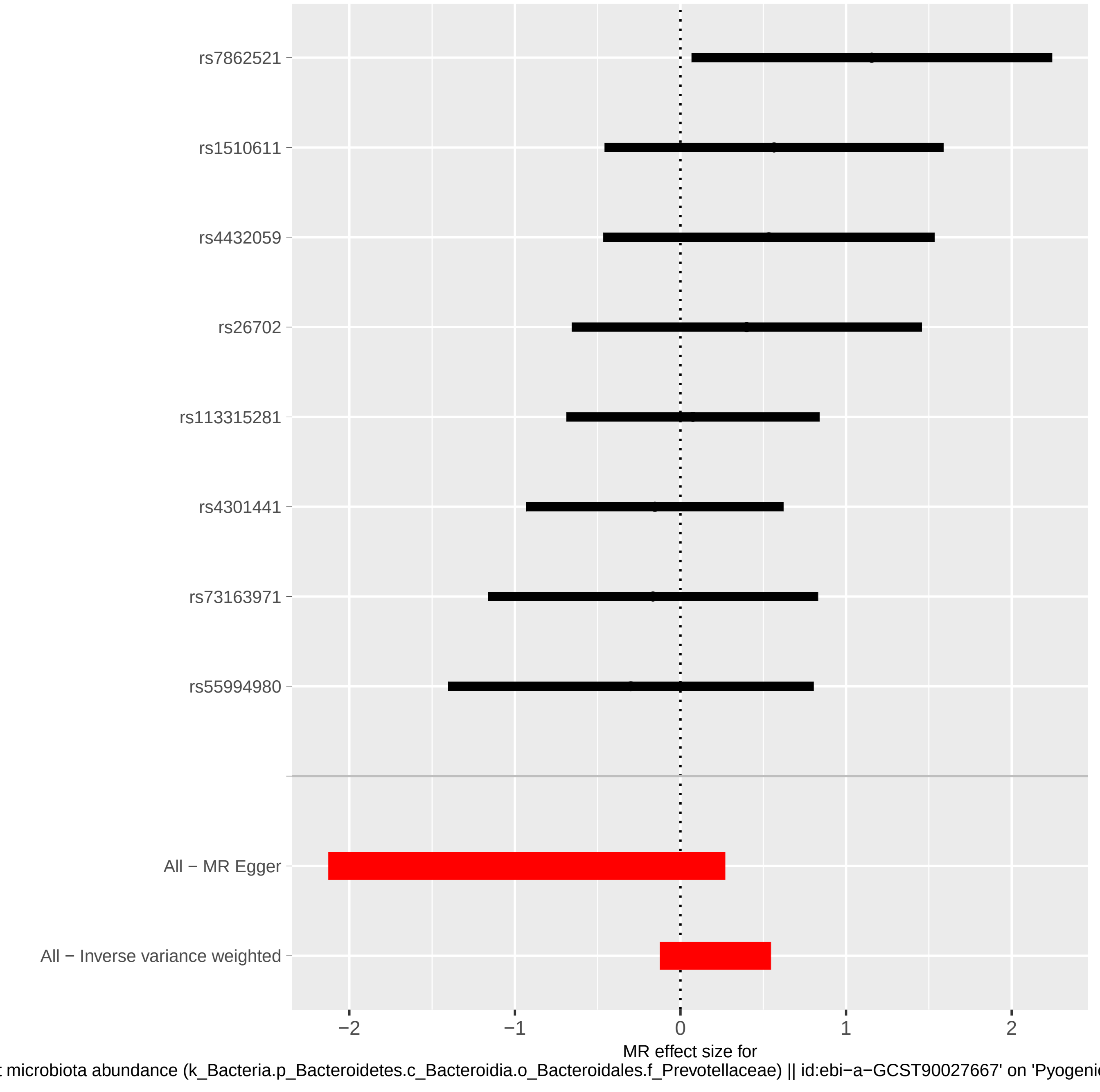

rs7862521
rs1510611
rs4432059
rs26702
rs113315281
rs4301441
rs73163971
rs55994980
All − MR Egger
All − Inverse variance weighted
−2
−1
0
1
2
MR effect size for
t microbiota abundance (k_Bacteria.p_Bacteroidetes.c_Bacteroidia.o_Bacteroidales.f_Prevotellaceae) || id:ebi−a−GCST90027667' on 'Pyogenic
